# Single-Fluorescence ATP Sensor Based on Fluorescence Resonance Energy Transfer Reveals Role of Antibiotic-Induced ATP Perturbation in Mycobacterial Killing

**DOI:** 10.1128/msystems.00209-22

**Published:** 2022-05-26

**Authors:** Lujie Liang, Daixi Lin, Yishen Chen, Jiachen Li, Wanfei Liang, Hui Zhao, Wenji Luo, Guo-bao Tian, Siyuan Feng

**Affiliations:** a Department of Microbiology, Zhongshan School of Medicine, Sun Yat-sen University, Guangzhou, China; b Key Laboratory of Tropical Diseases Control (Sun Yat-sen University), Ministry of Education, Guangzhou, China; c Guangdong Provincial Key Laboratory of Biotechnology for Plant Development, School of Life Sciences, South China Normal University, Guangzhou, China; d Department of Pharmacy, The Fifth Affiliated Hospital of Sun Yat-sen University, Zhuhai, China; Southern Medical University

**Keywords:** ATP, FRET, *Mycobacterium*, drug combination

## Abstract

The rapid emergence of multidrug-resistant/extensively drug-resistant tuberculosis (TB) is responsible for treatment failure in patients with TB and significantly endangers global public health. Recently, bioenergetics has become a new paradigm for anti-TB drug discovery and is based on the link between bacterial ATP levels and drug efficacy. A better understanding of the role of ATP fluctuations during antibiotic treatment may provide insight into antibiotic-mediated killing of mycobacteria. Here, we employed an advanced single-fluorescence FRET (fluorescence resonance energy transfer)-based ATP biosensor, ATPser, for the stable and convenient detection of intracellular ATP fluctuations in mycobacteria. This strategy correlated closely with the results obtained from conventional luminescence ATP assays, indicating the reliability of the system for bioenergetics analysis in mycobacteria. Moreover, the reporter strains expressing ATPser displayed obvious ATP changes when subjected to different stresses, such as starvation and ATP depletion. Interestingly, we observed that different antibiotics induced fluctuations in cellular ATP levels in individual cells of various magnitudes, revealing a strong connection between ATP fluctuations and drug efficacy. Furthermore, drug combinations accelerated ATP perturbation, resulting in increased cell death. We concluded that ATPser enabled real-time measurement of ATP at the single-cell level in mycobacteria, and monitoring ATP dynamics in drug-treated bacteria may shed light on novel treatment strategies.

**IMPORTANCE** Bioenergetics has emerged as a new paradigm for antituberculosis (anti-TB) drug discovery, and the cellular ATP level is the core indicator reflecting bacterial metabolic homeostasis. Although several bulk assays have been designed for the measurement of cellular ATP content, a more convenient strategy is required for real-time ATP measurement of single viable cells. In this study, by combining the ε-subunit of Bacillus subtilis F_o_F_1_-ATP synthase with a circularly permuted green fluorescent protein [(cp)GFP], we constructed a FRET-based single-fluorescence ATP sensor, ATPser, for real-time single-cell ATP detection among a mycobacterial population. Using the ATPser, we designed different drug combinations containing components that have similar/opposite effects on ATP alternation. Our results demonstrated that increased cellular ATP fluctuations were associated with depletion of mycobacterial viability, while counteracting ATP fluctuations weakened the killing effect of the drug regime. Thus, potentially efficient drug combinations can be considered based on their similar effects on mycobacterial ATP levels, and ATPser may be a useful tool to study mycobacterial bioenergetics and to guide drug regime design.

## INTRODUCTION

Mycobacterium tuberculosis causes a range of continuous lethal infections in humans. Based on the 2020 Global Tuberculosis Report from the World Health Organization (WHO), 10 million people were affected by tuberculosis (TB) and approximately 1.2 million died from TB that year (1). TB is therefore a great threat to global public health; however, the number of cases gradually decreased at a rate of 1.6%, on average, per year from 2000 to 2018 (2). Despite this, the emergence of multidrug-resistant (MDR) TB and extensively drug-resistant (XDR) TB has significantly limited the effectiveness of first/second-line anti-TB drugs (3). More toxic and expensive alternative drugs are therefore required for patients with MDR/XDR TB (4). Furthermore, unlike drug-sensitive TB, which can be cured within 6 months, longer durations of therapy (18 to 24 months) are required for MDR/XDR TB (5), often resulting in severe side effects or even treatment failure (6). Thus, novel anti-TB drugs and drug regimens are urgently needed to address this major clinical challenge.

Recently, bioenergetics has emerged as a field of interest for mycobacterial research and a new paradigm for anti-TB drug discovery (7, 8), highlighting the critical role of intracellular ATP in mycobacterial killing. For example, bedaquiline (BDQ) (9), Q203 (10), SQ109 (11), and delamanid (12) are new suppressors targeting a range of enzymes or cofactors responsible for the mycobacterial electron transport chain that stop oxidative phosphorylation and block energy generation in mycobacteria. These chemotherapeutics suppress cellular ATP formation and therefore appear to be capable of killing dormant mycobacteria, unlike conventional chemotherapeutics (13, 14).

The cellular ATP content is an indicator of the physiological state of bacteria (15), especially regarding the relationship between cell viability and antibiotic efficacy. Acting as the core component of cellular metabolism, ATP is essential for the growth and proliferation of bacteria. Besides supporting energy for current bioprocesses, ATP also reflects bacterial behavior relating to the stress response and tolerance toward antibiotics (15–17). Changes in cellular ATP levels after antibiotic challenge also correlate with proliferation ability and cell membrane permeability breakdown in mycobacteria. Isolates maintaining high ATP levels resume growth and proliferation after antibiotic treatment, while isolates with low ATP levels fail to resume growth and tend to lyse (18). In addition, cellular ATP level fluctuations determine the switch between persister and drug-sensitive cell types (19–21). For example, in Escherichia coli, the depletion of cellular ATP promotes protein aggregation as part of the stress response, resulting in dormancy, while increased cellular ATP promotes resuscitation of antibiotic-tolerant cells (20). Moreover, antibiotic treatment drives bacterial ATP fluctuations to various magnitudes (18). For example, a mycobacterial cell wall synthesis inhibitor causes a lethal ATP burst, which is thought to be related to enhancement of the envelope stress response (22). Furthermore, the cellular ATP level is a more accurate predictor of antibiotic efficacy than growth rate, and high cellular ATP levels are likely linked with the high efficacy of antibiotics (23).

Several assays have been designed for detecting the ATP content of a mycobacterial population. The most common strategy for bulk measurement is the CellTiter-Glo luminescence assay (Promega), which involves cell lysis and a reaction between extracellular luciferin and ATP (24), but it is not suitable for real-time measurement of viable cells. Other strategies have been employed to evaluate the bacterial metabolic rate, which is regarded as an indirect evaluation of ATP generation. For example, the Seahorse XF analyzer (Agilent) can determine the bacterial real-time respiration rate in a bulk manner by measuring the oxygen consumption rate and medium acidification (25, 26). Recently, several pioneering researchers developed a series of fluorescence resonance energy transfer (FRET)-based cellular ATP biosensors for the real-time detection of single-cell ATP levels (27–29). The existing ATP biosensors utilize mainly the F_o_F_1_-ATP synthase ε-subunit from Bacillus subtilis for ATP binding. Cyan fluorescent protein (CFP) and yellow fluorescent protein (YFP) are employed for signal collection, where CFP acts as a donor and YFP acts as a receptor for energy transfer. While calculation of the CFP/YFP ratio reveals the ATP level of each single cell (28, 30), such biosensors composed of two fluorescent proteins are limited in their quantitative capacity (29) because the extenuated tail at the right end of the CFP emission spectrum limits the measurement efficiency of the system (31). Additionally, the maturation time lag for the two fluorescence detections and fusion protein degradation can lead to bias in the overall signals (29).

Here, we applied a single green fluorescent protein (GFP)-based genetically encoded ATP biosensor, designated ATPser, in mycobacteria for single-cell ATP detection. This strategy was convenient and reliable for mycobacterial ATP detection. We demonstrated that the sensor was sensitive enough to reflect cellular ATP switching caused by different environmental stresses. Moreover, individual Mycobacterium smegmatis mc^2^ 155 ATPser (WT_ATPser) cells treated with antibiotics could undergo cellular ATP fluctuations with different kinetics. Finally, ATPser facilitated the relationship between antibiotic efficacy and cellular ATP perturbation caused by various drug combinations, suggesting the role of antibiotic-induced ATP perturbation in mycobacterial killing. We concluded that the cellular ATP level could serve as an indicator for drug regime efficacy and that ATPser could provide guidance for mycobacterial therapeutic regime design.

## RESULTS

### Construction of a cellular ATP level biosensor in Mycobacterium.

**FIG 1:**
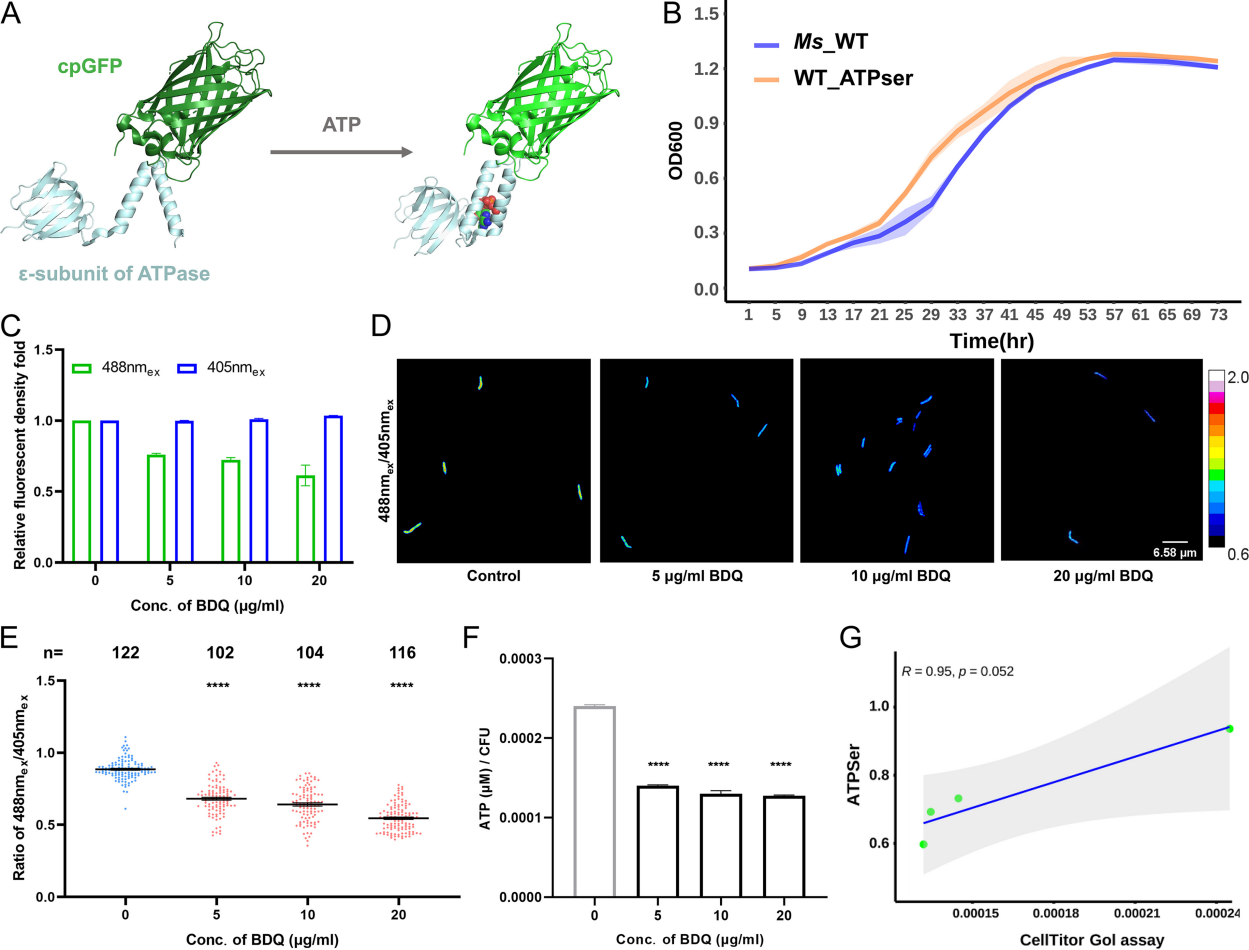
Construction of a genetically encoded FRET-based intracellular ATP biosensor in mycobacteria. A reporter strain, WT_ATPser, was constructed to reflect a dynamic change of cellular ATP level in M. smegmatis. (A) Stereo view of the ATPser complex protein, containing the cpGFP and the F_o_F_1_-ATP synthase ε-subunit from Bacillus subtilis. (Left) Natural form of the sensor; (right) interaction with ATP. The N-terminal β-strands are colored in green, and C-terminal α-helices are colored in light blue. The ATP is represented as a sphere model. Both the protein structure simulation and image plotting were processed by PyMOL (version 2.5). (B) Influence of ATPser expression on mycobacterial growth, where the growth curve of M. smegmatis WT carrying an empty plasmid (WT) is in purple and the growth curve of M. smegmatis expressing ATPser (WT_ATPser) is in orange. The growth curves were measured every 4 h, and the cell density at each time point is represented as the OD_600_ value. The graph was plotted by RStudio (version 3.6.1). (C) Sensitivity of fluorescence density for WT_ATPser with bedaquiline (BDQ) treatment. Logarithmic-phase culture was treated with 0, 5, 10, and 20 μg/mL BDQ for 2 h, and the fluorescence densities of 488 nm_ex_ and 405 nm_ex_ were measured by fluorescence microscopy and processed by Fiji. The bar graph shows the relative fluorescent density fold increase with BDQ treatment at different concentrations, compared with that of the untreated control, with the fluorescent density fold for 488 nm_ex_ shown in green and that for 405 nm_ex_ shown in blue. The graph was plotted by Prism 8. (D) Pseudocolored images showing the 488 nm_ex_/405 nm_ex_ ratio for WT_ATPser with BDQ treatment at the series of concentrations mentioned above. (E) Scatterplot of the 488 nm_ex_/405 nm_ex_ ratio for each individual bacterium. Each dot represents the ATP level in an individual bacterium. The untreated control is colored in blue, and the samples treated with BDQ are in red. *n*, number of isolates selected for analysis. The graph was plotted by Prism 8. (F) To confirm the reliability of ATPser, the average ATP level of the bacterial population described for panel C was confirmed by the CellTiter-Glo ATP bulk assay. The logarithmic-phase culture was selected for BDQ treatment at concentrations of 0, 5, 10, and 20 μg/mL for 2 h. The average cellular ATP level was measured using the CellTiter-Glo 2.0 assay kit (Promega). (G) The change in average ATP level was similar to that indicated by ATPser in panel E, the Pearson correlation coefficient between the results obtained from ATPser and the CellTiter-Glo ATP assay was calculated, and the graph was plotted by RStudio (version 3.6.1). The *R* value is 0.95, which indicates high consistency of the results obtained from the two assays. The pseudocolored images in panel D was processed and plotted by Fiji, and the color scale for the ratio values indicates high and low intracellular ATP levels. The bar graphs of panel F was plotted by Prism 8, where the control group is in gray and samples treated with antibiotics are in black. All the above-described experiments were performed three times with similar results. Error bars indicate standard errors of the means (SEM) for three biological replicates. A two-tailed unpaired *t* test was performed to determine the statistical significance of the data. ns, no significant difference; *, *P* < 0.1; **, *P* < 0.01; ***, *P* < 0.001; ****, *P* < 0.0001.

We aimed to generate a genetically encoded intracellular ATP sensor to reflect real-time ATP fluctuations in a single cell. This ratiometric ATP biosensor, designated ATPser, was composed of a circularly permuted enhanced green fluorescent protein (cpEGFP) that was inserted between two α-helices of the F_o_F_1_-ATP synthase ε-subunit from B. subtilis and connected by two amino acid linkers (Fig. 1A). This strategy has previously been adapted to create the FRET-based ATP sensors QUEEN (29) and the derivative iATPSnFr^1.0^ (27). This sensor has medium affinity for ATP (50% effective concentration [EC_50_] of 350 mM ATP with a maximum ATP-dependent fluorescence intensity increases d*F/F* of 1.0 in vivo, where dF/*F* was defined as (F(t) - F_0_)/F_0_ and F(t) is the fluorescent value after the addition of ATP and F_0_ is the initiation fluorescence value) (27), which is within the range of cytoplasmic ATP levels. The codon-optimized ATPser was expressed in M. smegmatis mc^2^ 155 from an L5-based integrating plasmid and controlled by a constitutive promoter. No growth defects were observed for WT_ATPser in comparison with M. smegmatis mc^2^ 155 carrying pMV306 empty plasmid (M. smegmatis WT) (Fig. 1B; see Fig. S3 in the supplemental material).

10.1128/msystems.00209-22.3 FIG S3 Growth rates for M. smegmatis wild type (WT) and WT_ATPser during exponential phase. The bar graphs represent the average growth rates of the two strains from 9 to 45 h as described in the legend for Fig. 1G. Cell density was represented by OD_600_ and measured every 4 h, and the growth rate between two neighbor time points was calculated. The average growth rate from 9 to 45 h was regarded as the growth rate during exponential phase. Error bars indicate the SEM for three biological replicates, and each point represents one biological repeat. Download FIG S3, TIF file, 0.02 MB. Copyright © 2022 Liang et al. 2022 Liang et al. https://creativecommons.org/licenses/by/4.0/ This content is distributed under the terms of the Creative Commons Attribution 4.0 International license.

**FIG 2:**
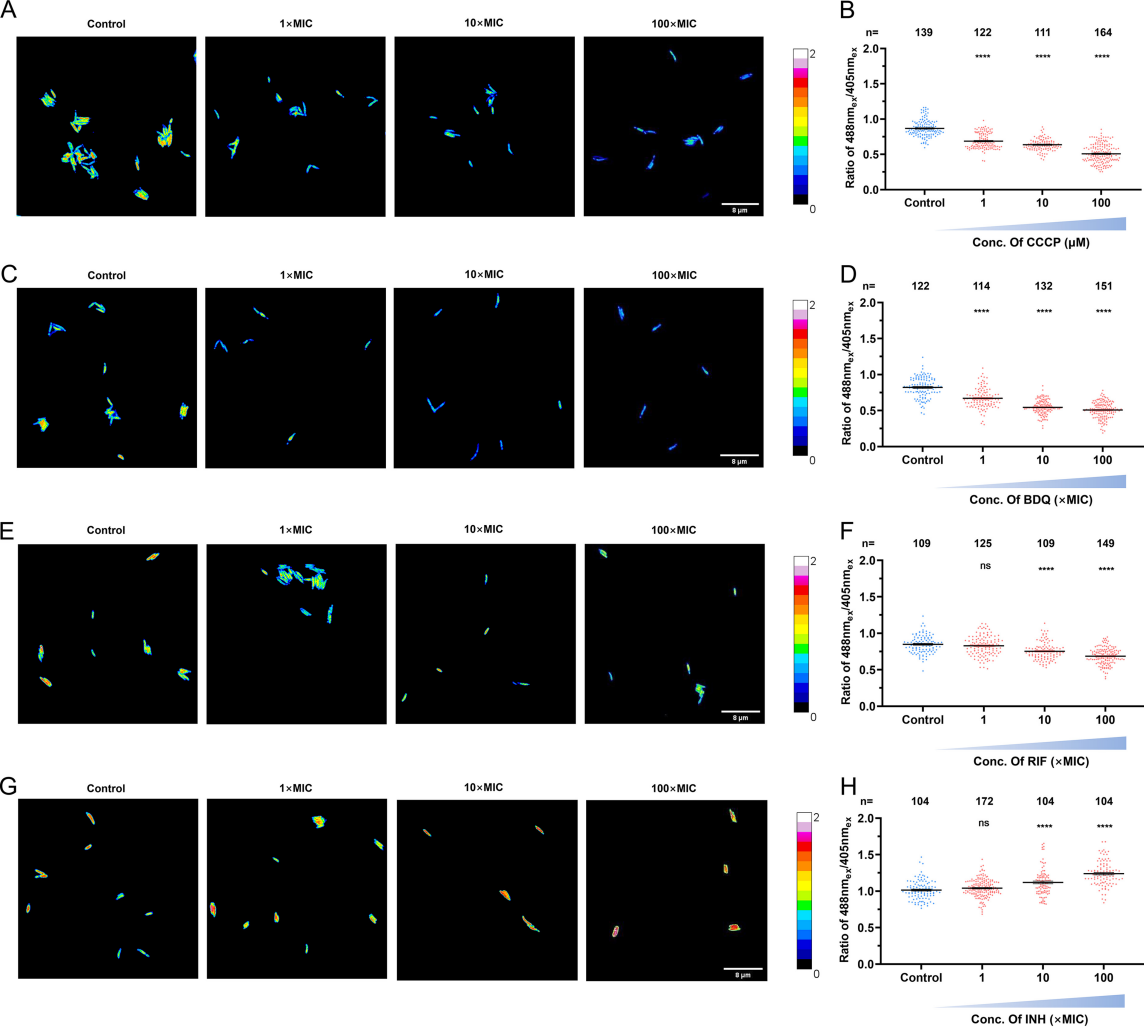
Application of ATPser in M. tuberculosis. Reporter strain M. tuberculosis mc^2^ 6206 pATPser was treated with different drugs to clarify the sensitivity of ATPser in M. tuberculosis. (A and B) To reduce the cellular ATP level of the reporter strain, the logarithmic-phase culture was treated with 1 μM, 10 μM, and 100 μM CCCP for 12 h. (A) Pseudocolored images of the 488 nm_ex_/405 nm_ex_ ratio for M. tuberculosis mc^2^ 6206 with treatments of 0 μM, 1 μM, 10 μM, and 100 μM CCCP. (B) Scatterplot of the 488 nm_ex_/405 nm_ex_ ratio for each individual as described for panel A. To verify the influence of bedaquiline (BDQ) on the cellular ATP level, the logarithmic-phase M. tuberculosis mc^2^ 6206 culture was treated with 0 μg/mL (control), 0.28 μg/mL (1× MIC), 2.8 μg/mL (10× MIC), and 28 μg/mL (100× MIC) BDQ for 12 h. (C) Pseudocolored images of the 488 nm_ex_/405 nm_ex_ ratio for WT_ATPser with treatments of 0×, 1×, 10×, and 100× MIC BDQ. (D) Scatterplot of the 488 nm_ex_/405 nm_ex_ ratio for each individual as described for panel C. To verify the influence of rifampin (RIF) on the cellular ATP level, the logarithmic-phase M. tuberculosis mc^2^ 6206 culture was treated with 0 μg/mL (control), 0.0823 μg/mL (1× MIC), 0.823 μg/mL (10× MIC), and 8.23 μg/mL (100× MIC) RIF for 12 h. (E) Pseudocolored images of the 488 nm_ex_/405 nm_ex_ ratio for WT_ATPser with treatments of 0×, 1×, 10×, and 100× MIC BDQ. (F) Scatterplot of the 488 nm_ex_/405 nm_ex_ ratio for each individual as described for panel E. To verify the influence of isoniazid (INH) on the cellular ATP level, the logarithmic-phase M. tuberculosis mc^2^ 6206 culture was treated with 0 μg/mL (control), 0.5 μg/mL (1× MIC), 5 μg/mL (10× MIC), and 50 μg/mL (100× MIC) INH for 12 h. (G) Pseudocolored images of the 488 nm_ex_/405 nm_ex_ ratio for WT_ATPser with treatments of 0×, 1×, 10×, and 100× MIC INH. (H) Scatterplot of the 488 nm_ex_/405 nm_ex_ ratio for each individual as described for panel G. The pseudocolored images in panels A, C, E, and G were processed and plotted by Fiji, and the color scale for the ratio values indicates high and low intracellular ATP levels. The scatterplots of panels B, D, F, and H were plotted by Prism 8, where *n* represents the number of isolates selected for analysis. The control group is shown in blue, and the groups with drug treatments are shown in red. All of the experiments described above were performed three times with similar results. Error bars indicate the SEM for three biological replicates. A two-tailed unpaired *t* test was performed to determine the statistical significance of the data. ns, no significant difference; ****, *P* < 0.0001.

The ε-subunit of F_o_F_1_-ATP synthase from B. subtilis consists of eight N-terminal β-strands followed by two α-helices located at the C terminus, which has specific affinity toward cellular ATP (32). In the absence of ATP, the ε-subunit was flexible and two α-helices were extended. When ATPser binds with ATP, the ε-subunit retracts to draw the two α-helices close to each other (Fig. 1A). The reaction changes the structure of the fusion protein and influences the protonation/deprotonation of cpEGFP (29, 33). The cpEGFP shows excitation at wavelengths of 405 nm and 488 nm and emits at a wavelength of 515 nm, and the protonation/deprotonation can cause further fluctuation of the fluorescence under excitation at 405-nm or 488-nm wavelengths. The ratio between the fluorescent signals from the two excitation wavelengths (488 nm_ex_/405 nm_ex_) is calculated to reflect the ATP concentration of each single cell. To evaluate the influence of cellular ATP on the fluorescence at the two excitation wavelengths and the 488 nm_ex_/405 nm_ex_ ratio, we treated WT_ATPser with increasing concentrations of BDQ, an anti-TB drug targeting the F_o_F_1_-ATP synthase of mycobacteria and inhibiting the generation of cellular ATP. As for the change in fluorescent intensity, the addition of BDQ resulted in a decrease in the 488 nm_ex_ value and an increase in the 405 nm_ex_ value (Fig. 1C; Fig. S4), leading to a shift to a lower 488 nm_ex_/405 nm_ex_ ratio for each individual cell under high-concentration BDQ treatment (Fig. 1E). The 488 nm_ex_/405 nm_ex_ ratio is displayed as a calibration color series to reflect cellular ATP qualitatively, and BDQ treatment evidently shifted the artificial color of each single cell from green/yellow to blue (Fig. 1D), indicating a drop in cellular ATP. We also performed the same experiment by bulk measurement of the intracellular ATP level of the WT_ATPser population with the commercial CellTiter-Glo ATP assay to confirm the results obtained with ATPser. Consistent with the observations from fluorescence imaging, a significant drop in the ATP level after BDQ treatment was also observed in this assay (Fig. 1F). Moreover, the strong correlation (*R *= 0.95) (Fig. 1G) between the results from the two assays revealed that ATPser indeed reflects cellular ATP depletion caused by BDQ inhibition. Moreover, an M. tuberculosis mc^2^ 6206 ATP reporter strain was also constructed and treated with CCCP (carbonyl cyanide *m*-chlorophenylhydrazone; an ATP synthase inhibitor), BDQ, rifampin (RIF), and isoniazid (INH) in a concentration series to test the sensitivity of ATPser in M. tuberculosis. By calculating the 488 nm_ex_/405 nm_ex_ ratio, we found that after CCCP (Fig. 2A and B) or BDQ (Fig. 2C and D) treatment, the intracellular ATP level of M. tuberculosis decreased in a dose-dependent manner. Treatment with 1× MIC RIF had no significant influence on mycobacterial ATP level in comparison with that of the control, while treatment with 10× or 100× MIC RIF led to a significant decrease of the 488 nm_ex_/405 nm_ex_ ratio (Fig. 2E and F). Also, treatment with 1× MIC INH had no significant influence on cellular ATP levels, but 10× or 100× MIC INH treatment resulted in a significant increase in *in vivo* ATP levels (Fig. 2G and H). These results further confirmed that the ATP sensor generated in this study was reliable and could be applied to M. tuberculosis. Above all, our results demonstrated that ATPser was a precise sensor for tracking real-time cellular ATP fluctuations in mycobacteria.

10.1128/msystems.00209-22.4 FIG S4 Fluorescence intensity of WT_ATPser treated with bedaquiline (BDQ). The bar graph shows the fluorescence intensity of WT_ATPser with treatments with BDQ at different concentrations, as described in the legend for Fig. 1C, which exhibited the average gray values of three repeats under each treatment, each point representing one repeat. Error bars indicate the SEM for three biological replicates. The graph was plotted by Prism 8. Download FIG S4, TIF file, 0.2 MB. Copyright © 2022 Liang et al. 2022 Liang et al. https://creativecommons.org/licenses/by/4.0/ This content is distributed under the terms of the Creative Commons Attribution 4.0 International license.

### ATPser indicates cellular ATP fluctuations during various stresses.

**FIG 3:**
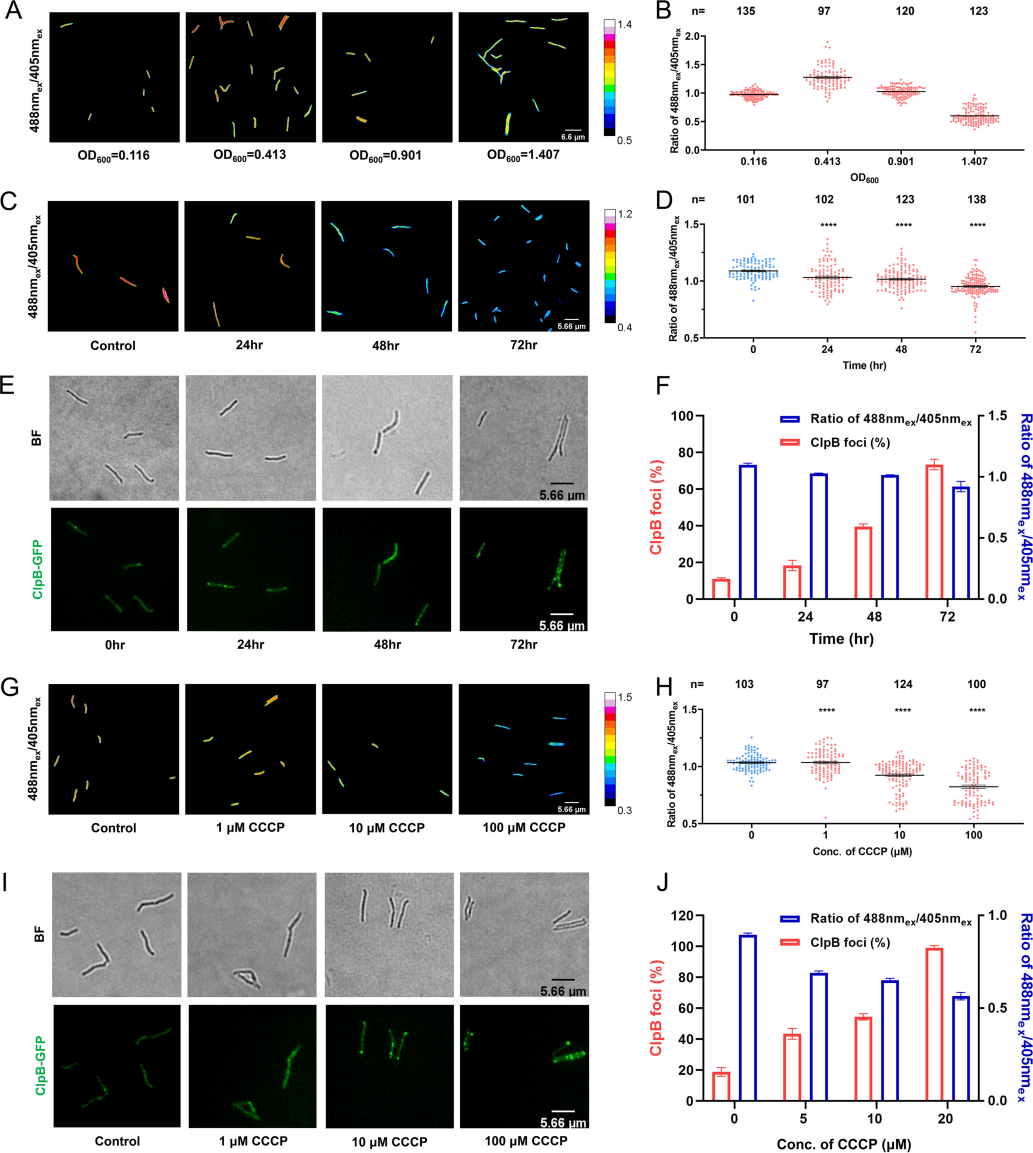
ATPser reflecting cellular ATP level fluctuation related to formation of ClpB foci. Several assays were applied to verify the influence of environmental stress on the WT_ATPser cellular ATP level. (A and B) Change in cellular ATP level for WT_ATPser during cultivation in rich medium. Logarithmic-phase culture was utilized, and the cell density was adjusted to an OD_600_ of 0.4 for the seed culture. The seed culture was subcultured into fresh 7H9 medium at a ratio of 1:100, and the fluorescence densities of 488 nm_ex_ and 405 nm_ex_ for the WT_ATPser reporter strain were measured by fluorescence microscopy at different growth phases. (A) Pseudocolored images showing the 488 nm_ex_/405 nm_ex_ ratio for WT_ATPser during cultivation in rich medium at lag phase (OD_600_ = 0.116), log phase (OD_600_ = 0.413), stationary phase (OD_600_ = 0.901), and death phase (OD_600_ = 1.407), indicating change of cellular ATP level during different growth phases. (B) Scatterplot of the 488 nm_ex_/405 nm_ex_ ratio for each individual as described for panel A. (C and D) Influence of starvation treatment on cellular ATP level dynamic change. Logarithmic-phase WT_ATPser culture was collected and treated with PBST to mimic poor nutrition stress. The fluorescence densities of 488 nm_ex_ and 405 nm_ex_ for the reporter strain were measured by fluorescence microscopy every 24 h. (C) Pseudocolored images of the 488 nm_ex_/405 nm_ex_ ratio for the reporter strain at 0, 24, 48, and 72 h after PBST treatment. (D) Scatterplot of the 488 nm_ex_/405 nm_ex_ ratio for each individual as described for panel C. To verify the correlation between cellular stress response caused by poor nutrition and cellular ATP dynamic change, a percentage of ClpB foci for the WT_ClpB_EGFP reporter strain was treated with PBS as described for panels C and D. (E) The samples were subjected to fluorescence microscopy, and bright-field (BF) microscopy and FITC staining images were captured every 24 h, showing the formation of ClpB foci and indicating the cellular stress response during poor nutrition stress. (F) Cell fraction with ClpB foci for the WT_ClpB_EGFP reporter strain and the average 488 nm_ex_/405 nm_ex_ ratio for WT_ATPser at 0, 24, 48, and 72 h after PBST treatment. The increase in the cell fraction with ClpB foci (red bars) is accompanied by depletion of the average cellular ATP level (blue bars) after PBST treatment. To verify the correlation between cellular stress response and ATP depletion, the fractions of cells with ClpB foci for WT_ClpB_EGFP and the 488 nm_ex_/405 nm_ex_ ratio for WT_ATPser were measured with the treatment of CCCP. Logarithmic-phase WT_ATPser culture was treated with CCCP. The fluorescence densities of 488 nm_ex_ and 405 nm_ex_ for the reporter strain were measured by fluorescence microscopy 4 h after CCCP treatment. (G) Pseudocolored images of the 488 nm_ex_/405 nm_ex_ ratio for WT_ATPser with CCCP treatment at concentrations of 0, 1, 10, and 100 μM. (H) Scatterplot of the 488 nm_ex_/405 nm_ex_ ratio for each individual as described for panel G. The samples of WT_ClpB_EGFP with CCCP treatment in panels G and H were subjected to fluorescence microscopy, and bright-field (BF) microscopy and FITC staining images were captured 4 h after treatment, as for panel E, and show the formation of ClpB foci and indicate the cellular stress response during CCCP treatment. (I) Cell fraction with ClpB foci for the WT_ClpB_EGFP reporter strain and the average 488 nm_ex_/405 nm_ex_ ratio for WT_ATPser with CCCP treatment at concentrations of 0, 1, 10 and 100 μM. With the increase in CCCP concentration, the cell fraction with ClpB (blue bars) increased and the average cellular ATP level (red bars) decreased. The pseudocolored images of panels A, C, and G were processed and plotted by Fiji, and the color scale for the ratio values indicate high and low intracellular ATP levels. The scatterplots of panels B, D, and H were plotted by Prism 8, where *n* represents the number of isolates selected for analysis. The bar graphs of panels F and J were plotted by Prism 8. All the above-described experiments were performed three times with similar results. Error bars indicate the SEM for three biological replicates. A two-tailed unpaired *t* test was performed to determine the statistical significance of the data. ****, *P* < 0.0001.

It is known that environmental fluctuations can influence the bacterial metabolic state, which is closely associated with the cellular ATP level. We hypothesized that the sensor generated in this study enabled the monitoring of changes in intracellular ATP levels in response to environmental fluctuations. To test this, we first measured cellular ATP levels throughout culturing in rich medium by collecting samples from different growth stages. As shown in Fig. 3A, the artificial color of cells changed from green/yellow to yellow/red at exponential phase, indicating an increase in cellular ATP. The cells that appeared yellow at early stationary phase and green at late stationary phase represented continuous depletion of ATP during this stage. The value of the 488 nm_ex_/405 nm_ex_ ratio displayed a similar pattern of cellular ATP fluctuation (Fig. 3B).

Bacteria can undergo protein aggregation as a protective response to environmental stress, with aggregated proteins mainly localizing to the two poles (20). Previous studies have shown that ClpB is an essential enzyme required for disaggregation, binding to the aggresome during the cellular stress response in both M. tuberculosis and E. coli (20, 34). Therefore, the enrichment of ClpB at the bacterial poles can reflect the existence of aggresomes, which are indicators of the cellular stress response. To further explore the relationship between the degree of stress and ATP fluctuation, we constructed a reporter strain, WT_ClpB_EGFP, to reflect the response to environmental stress. WT_ATPser and reporter strain WT_ClpB_EGFP were first cultivated with nutrient-rich medium to exponential phase, and then the medium was changed to phosphate-buffered saline with Tween 20 (PBST) and cultivation continued for 72 h with shaking. We observed that the intracellular ATP level was significantly decreased in a time-dependent manner (Fig. 3C and D). The results with ATPser were consistent with those of the CellTiter-Glo ATP assay (Fig. S5A). We also estimated the extent of the stress response by counting the ClpB foci. Compared with the sample collected at 0 h, the fraction of cells containing ClpB foci increased gradually during starvation stress (Fig. 3E and F) and was negatively correlated with the cellular ATP level (Fig. S6A) (*R* = −0.95). These results demonstrated that the decrease in cellular ATP levels measured by ATPser caused the stress response and aggregation of ClpB at the poles.

10.1128/msystems.00209-22.5 FIG S5 Measurement of bulk ATP level by CellTiter-Glo assay. The change in average ATP level for WT_ATPser was confirmed by the CellTiter-Glo assay. (A) Change in bulk ATP level for WT_ATPser cultivated in rich medium at different growth phases as described in the legend to Fig. 2A and B. (B) Change in bulk ATP level for WT_ATPser treated with CCCP in a series of concentrations as described in the legend to Fig. 2E and F, where the control group is in gray and samples treated with CCCP are in black. All the above-described experiments were performed three times with similar results. Error bars indicate the SEM for three biological replicates. A two-tailed unpaired *t* test was performed to determine the statistical significance of the data. ns, no significant difference; **, *P* < 0.01; ****, *P* < 0.0001. Download FIG S5, TIF file, 0.1 MB. Copyright © 2022 Liang et al. 2022 Liang et al. https://creativecommons.org/licenses/by/4.0/ This content is distributed under the terms of the Creative Commons Attribution 4.0 International license.

10.1128/msystems.00209-22.6 FIG S6 Correlations between the 488 nm_ex_/405 nm_ex_ ratio and the fraction of ClpB foci among the bacterial population. (A and B) Pearson’s correlation coefficient between the average value of the 488 nm_ex_/405 nm_ex_ ratio and the cell fraction of ClpB foci was calculated, and the graph was processed and plotted by RStudio (version 3.6.1). The correlation coefficient (*R*) for reporter strains during growth in rich medium as described in the legend to Fig. 2A and B is −0.95 (A), while the coefficient is −0.91 for reporter strains treated with CCCP as described in the legend to Fig. 2E and F (B). The high correlation coefficients indicate strong consistency between the mycobacterial stress response and the cellular ATP perturbation. Download FIG S6, TIF file, 0.05 MB. Copyright © 2022 Liang et al. 2022 Liang et al. https://creativecommons.org/licenses/by/4.0/ This content is distributed under the terms of the Creative Commons Attribution 4.0 International license.

In addition, we also mimicked cellular energy limitation stress by treating WT_ATPser with a series of concentrations of CCCP, a chemical inhibitor of oxidative phosphorylation that causes uncoupling of the proton gradient in the cell membrane electron transport chain and inhibits ATP synthesis (35). As shown in Fig. 3G and H, the cellular ATP level changed in a dose-dependent manner, in which the ATP level declined slightly for cells treated with 1 μM CCCP and reached its lowest level for cells treated with 100 μM CCCP, which was consistent with the results of the CellTiter-Glo ATP assay (Fig. S5B). Similar to starvation stress, the increase in CCCP concentration was accompanied by an increased fraction of cells containing ClpB foci (Fig. 3I and J), which were also negatively correlated with each other (Fig. S6B) (*R* = −0.91). We also found that there was no significant effect on mycobacterial viability from treatment with CCCP at concentrations ranging from 1 μM to 100 μM during the first 24 h. Whereas treatment with 100 μM CCCP resulted in a significant decrease in viability at 48 and 72 h, the remaining treatments maintained viability similar to that of the untreated control (Fig. S7). Taken together, these results confirmed that ATPser is a biosensor with high sensitivity for detecting cellular ATP changes during various types of stress that induce a significant cellular stress response in mycobacteria.

10.1128/msystems.00209-22.7 FIG S7 Changes in bacterial viability after treatment with CCCP. The bar graph reveals the viability of the reporter strain treated with CCCP. The logarithmic-phase culture of WT_ATPser was treated with 0, 1, 10, and 100 μM CCCP, and the viability was verified by CFU assay every 24 h after treatment. Error bars indicate the SEM for three biological replicates. A two-tailed unpaired *t* test was performed to determine the statistical significance of the data. ns, no significant difference; *, *P* < 0.1; ***, *P* < 0.001. Download FIG S7, TIF file, 0.03 MB. Copyright © 2022 Liang et al. 2022 Liang et al. https://creativecommons.org/licenses/by/4.0/ This content is distributed under the terms of the Creative Commons Attribution 4.0 International license.

### Monitoring the effects of anti-TB drugs on cellular ATP fluctuations using ATPser.

**FIG 4:**
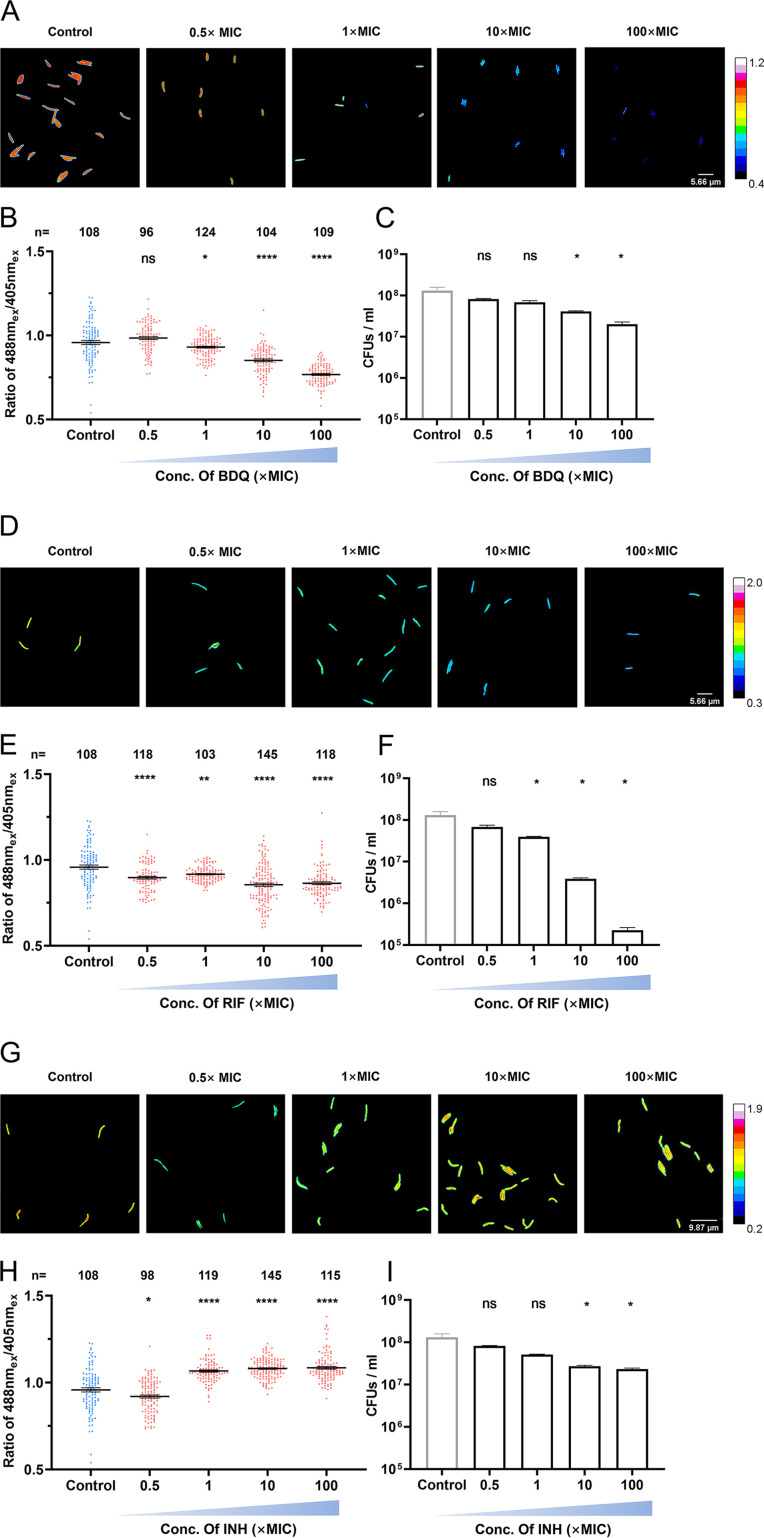
Discrimination of antibiotics leading to cellular ATP switching in different kinetics. Reporter strain WT_ATPser was treated with different antimycobacterial drugs to investigate the correlation between cellular ATP level change and viability. (A to C) To reduce the cellular ATP level of the reporter strain, the logarithmic-phase WT_ATPser culture was treated with 0 μg/mL (control), 0.025 μg/mL (0.5× MIC), 0.05 μg/mL (1× MIC), 0.5 μg/mL (10× MIC), and 5 μg/mL (100× MIC) bedaquiline (BDQ) for 4 h. (A) Pseudocolored images of the 488 nm_ex_/405 nm_ex_ ratio for WT_ATPser with treatment of 0, 0.5, 1, 10, and 100× MIC BDQ. (B) Scatterplot of the 488 nm_ex_/405 nm_ex_ ratio for each individual as described for panel A. (C) To verify the change in viability with BDQ treatment, a CFU assay was performed after drug treatment, and the viability was shown. (D) To verify the influence of rifampin (RIF) on cellular ATP level, the logarithmic-phase WT_ATPser culture was treated with 0 μg/mL (control), 4 μg/mL (0.5× MIC), 8 μg/mL (1× MIC), 80 μg/mL (10× MIC), and 800 μg/mL (100× MIC) RIF for 4 h. Pseudocolored images of the 488 nm_ex_/405 nm_ex_ ratio for WT_ATPser with treatment of 0×, 0.5×, 1×, 10×, and 100× MIC RIF are shown. (E) Scatterplot of the 488 nm_ex_/405 nm_ex_ ratio for each individual as described for panel D. (F) To verify the change in viability with RIF treatment, a CFU assay was performed after drug treatment, and the viability was shown. To increase the cellular ATP level of the reporter strain, logarithmic-phase WT_ATPser culture was treated with 0 μg/mL (control), 4 μg/mL (0.5× MIC), 8 μg/mL (1× MIC), 80 μg/mL (10× MIC), and 800 μg/mL (100× MIC) isoniazid (INH) for 4 h. (G) Pseudocolored images of the 488 nm_ex_/405 nm_ex_ ratio for WT_ATPser with treatment of 0×, 0.5×, 1×, 10×, and 100× MIC INH. (H) Scatterplot of the 488 nm_ex_/405 nm_ex_ ratio for each individual as described for panel G. (I) To verify the change in viability with INH treatment, a CFU assay was performed after drug treatment, and the viability was shown. The pseudocolored images of panels A, D, and G were processed and plotted by Fiji, and the color scale for the ratio values indicates high and low intracellular ATP levels. The scatterplots of panels B, E, and H were plotted by Prism 8, where *n* represents the number of isolates selected for analysis. The control group is in blue, and the groups with drug treatments are in red. The bar graphs of panels C, F, and I were plotted by Prism 8. The control group is in gray, and the groups with drug treatments are in black. All the above-described experiments were performed three times with similar results. Error bars indicate the SEM for three biological replicates. A two-tailed unpaired *t* test was performed to determine the statistical significance of the data. ns, no significant difference; *, *P* < 0.1; ****, *P* < 0.0001.

Accumulated evidence suggests that anti-TB drugs induce metabolic stress in mycobacteria (25), and antibiotics influence cellular bioenergetics, with differential kinetic profiles, due to various mechanisms (18). To assess the impact of antibiotics on cellular ATP levels, we selected three types of anti-TB drugs that target different essential molecules in mycobacteria: BDQ (F_o_F_1_-ATP synthase), RIF (RNA polymerase), and INH (fatty acid synthase). WT_ATPser was treated with the above-mentioned antibiotics at concentrations of 0.5×, 1×, 10×, and 100× MIC for 2 h, and the CFU were counted to calculate viability. With the increased concentration of BDQ, the ATP levels of the population and the viability decreased in a dose-dependent manner (Fig. 4B and C), and the artificial color changed from red in the untreated control to deep blue when the highest concentration of BDQ was administered (Fig. 4A). This result confirmed that BDQ has a dose-dependent effect on cellular ATP levels.

Interestingly, RIF conferred a slight but significant decrease in the cellular ATP levels of the population and the artificial color of individual cells remained green/blue despite an increasing RIF concentration (Fig. 4D and E), whereas viability showed a steady decline (Fig. 4F). This result indicates that RIF may have a small impact on cellular metabolism within a short period. Since RIF is a transcription inhibitor, it is logical that it did not significantly affect cellular ATP levels.

Unlike treatment with BDQ and RIF, INH treatment enhanced cellular ATP levels at high concentrations (1×, 10×, and 100× MICs) (Fig. 4G and H), which was consistent with previous work (22, 36). However, when the INH concentration was higher than 1× MIC, no significant increase in ATP levels of the population as a whole was observed, and the artificial color of individual cells changed from red/yellow for the untreated control to green/yellow for samples treated with 0.5× or 1× MIC INH and to red/yellow for samples treated with 10× or 100× MIC INH (Fig. 4G). Increased cellular ATP levels and a significant decline in viability were simultaneously observed in samples treated with 10× or 100× MIC INH (Fig. 4I). Consistent with this, INH can inhibit the synthesis of fatty acid chains, further increasing cell membrane permeability and cellular stress, which results in perturbation of the electron transport chain and a lethal respiration burst (36).

Together, our results indicate that anti-TB drugs, which lead to mycobacterial killing, mediate fluctuations in intracellular ATP to various magnitudes.

### Perturbation of cellular ATP homeostasis linked with the promotion of cell death.

Previous studies suggested that antibiotics can be functionally effective by disrupting the homeostasis of bacterial metabolism, which further leads to enhancement or depletion of cellular respiration (37). Given that ATP is the major product of cellular respiration, we therefore proposed that acceleration of cellular ATP perturbation may be related to the promotion of cell death and that cellular ATP perturbation can be a marker to indicate viability after anti-TB drug treatment. To verify our hypothesis, three types of drug combinations were designed to perturb cellular ATP levels to different magnitudes: (i) BDQ and CCCP, which are both chemicals that inhibit the synthesis of cellular ATP and lead to depletion of ATP levels; (ii) ethambutol (EMB) plus INH, which is a drug combination commonly used in clinical practice (EMB can induce an ATP burst in mycobacteria, as previously reported [22], while INH can also lead to an increase in ATP levels); and (iii) BDQ plus INH, which is a combination of antibiotics that, individually, have opposite effects on mycobacterial ATP levels. WT_ATPser cells were treated with the above-mentioned drug combinations for 2 h. Samples without treatment and those treated with each drug singularly were included as controls.

**FIG 5:**
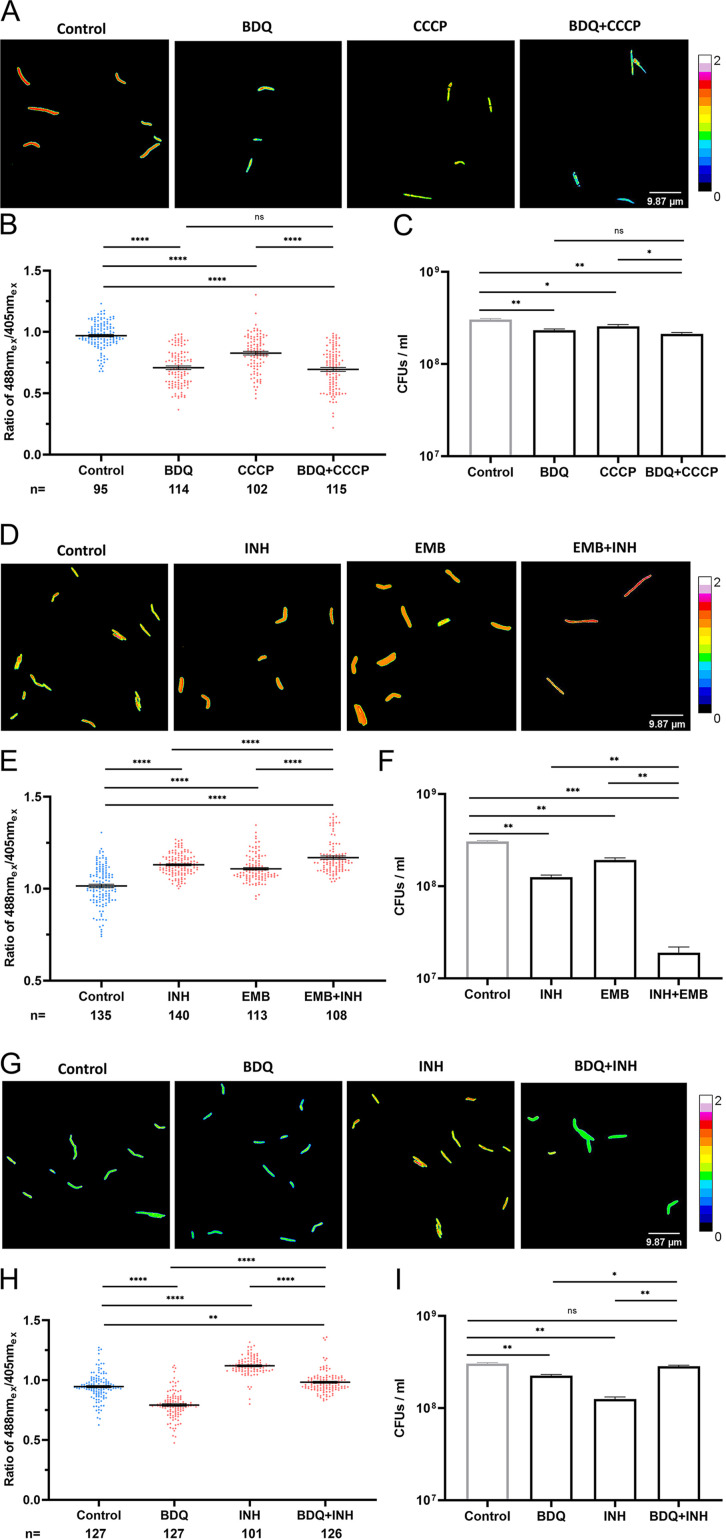
Effects of drug combinations on cellular ATP level and viability. Several drug combinations were designed to clarify the correlation between cellular ATP perturbation and mycobacterial viability. (A to C) To promote the decrease in cellular ATP level, logarithmic-phase WT_ATPser culture was treated with 10 μM CCCP, 0.5 μg/mL (10× MIC) BDQ, a regime containing the two drugs, and a culture without drug treatment (the control group). (A) Pseudocolored images of the 488 nm_ex_/405 nm_ex_ ratio for WT_ATPser with the treatments of BDQ, CCCP, and BDQ+CCCP. (B) Scatterplot of the 488 nm_ex_/405 nm_ex_ ratio for each individual as described for panel A. (C) To verify the change in viability with BDQ, CCCP, and BDQ+CCCP treatments, a CFU assay was performed after drug treatment, and the viability was shown. (D to F) To enhance the increase in cellular ATP level of the reporter strain, logarithmic-phase WT_ATPser culture was treated with 80 μg/mL (10× MIC) INH, 10 μg/mL (10× MIC) EMB, and a regime containing the two drugs. Culture without drug treatment was set as the control group. (D) Pseudocolored images of the 488 nm_ex_/405 nm_ex_ ratio for WT_ATPser with the treatments of INH, EMB, and INH+EMB. (E) Scatterplot of the 488 nm_ex_/405 nm_ex_ ratio for each individual as described for panel D. (F) To verify the change in viability with INH, EMB, and INH+EMB treatments, a CFU assay was performed after drug treatment, and the viability was shown. The WT_ATPser reporter strain was also treated with two antibiotics exhibiting opposite effects on the mycobacterial ATP level. Logarithmic-phase WT_ATPser culture was treated with 0.5 μg/mL (10× MIC) BDQ, 80 μg/mL (10× MIC) INH, and a regime containing the two drugs, and a culture without drug treatment was set as the control group. (G) Pseudocolored images of the 488 nm_ex_/405 nm_ex_ ratio for WT_ATPser with the treatments of BDQ, INH, and BDQ+INH. (H) Scatterplot of the 488 nm_ex_/405 nm_ex_ ratio for each individual as described for panel G. (I) To verify the change in viability with INH, EMB, and INH+EMB treatments, a CFU assay was performed after drug treatment, and the viability was shown. The pseudocolored images of panels A, D, and G were processed and plotted by Fiji, and the color scale for the ratio values indicates high and low intracellular ATP levels. The scatterplots of panels B, E, and H were plotted by Prism 8, where *n* represents the number of isolates selected for analysis. The control group is in blue, and the groups with drug treatments are in red. The bar graphs of panels C, F, and I were plotted by Prism 8. The control group is in gray, and the groups with drug treatments are in black. All the above-described experiments were performed three times with similar results. Error bars indicate the SEM for three biological replicates. A two-tailed unpaired *t* test was performed to determine the statistical significance of the data. ns, no significant difference; *, *P*< 0.1; **, *P* < 0.01; ****, *P* < 0.0001.

In the case of the BDQ+CCCP combination treatment, single treatments with BDQ and CCCP led to a sharp decline in cellular ATP levels, while the combination of the two drugs further decreased cellular ATP levels (Fig. 5A and B). Meanwhile, the viability of the sample treated with BDQ+CCCP was slightly lower than those of samples treated with the single drugs (Fig. 5C). These results revealed that BDQ and CCCP have synergistic effects on cellular ATP depletion and that the enhanced loss of mycobacterial ATP may be related to reduced viability.

EMB and INH are first-line antibiotics that have synergistic effects in the treatment of TB (38). As shown in Fig. 5D and E, both EMB and INH increased the cellular ATP level of the population significantly, while cotreatment with the two drugs further increased the ATP level compared with that of the sample treated with EMB alone. Furthermore, the acceleration of intracellular ATP accumulation was accompanied by a significant decline in viability (Fig. 5F), indicating that the synergistic effect of INH and EMB on cellular ATP acceleration correlates with increased efficacy.

Although both INH and BDQ have significant effects on cellular ATP fluctuations, no obvious switching of the ATP level was observed when these two drugs were used to treat WT_ATPser simultaneously (Fig. 5G and H), and the artificial color for each individual cell remained red/yellow in comparison with that of the untreated control (Fig. 5G). Nevertheless, single-drug treatment with INH or BDQ caused a decrease in viability, but cotreatment had no effect on cell survival in comparison with that of the untreated control (Fig. 5I). Obviously, the ATP burst elicited by INH was suppressed by BDQ, which further attenuated the lethal effect of the two drugs and revealed an antagonistic relationship between them.

In brief, our research revealed that the accelerated intracellular ATP fluctuations induced by different drug combinations are potentially associated with increased bacterial cell death.

## DISCUSSION

In this study, we developed an approach for single-cell real-time ATP level measurement in mycobacteria. We employed cpGFP as an indicator, and this biosensor effectively reflects cellular ATP levels in the 488 nm_ex_/405 nm_ex_ ratio for each individual cell among a population, while the conventional luminescence assay can detect only the bulk ATP content. Moreover, the results obtained with ATPser were consistent with those obtained by the luminescence assay, but ATPser was able to monitor the cellular ATP variation in individual cells in an accurate manner. Although the CFP/YFP-based ATP biosensor has previously been used in mycobacteria, the maturation time lag and the rarely used fluorescent proteins limited its application (29). By replacing the rarely used fluorescent proteins with versatile GFP, the FRET-based ATP sensor developed in the current study offered more stable detection and easier analysis (21, 27, 29). Our biosensor is, therefore, a potentially useful tool to study mycobacterial bioenergetics and the stress response.

In our research, we found that depletion of the cellular ATP level induced by starvation or inhibition of ATP synthase (using CCCP) was accompanied by an increased population carrying ClpB foci, indicating an enhanced stress response. In fact, it is known that the depletion of intracellular ATP is accompanied by an increased stress response, and there is a critical relationship between cellular ATP fluctuations and the stress response. In the cases of E. coli and Staphylococcus aureus, suppression of ATP synthesis stimulated by the aggregation of cellular protein further results in the generation of persisters that can resist antibiotics (20, 39). Although many studies considered the aggresome a toxic cellular component (40), the formation of an aggresome can also be considered a protective mechanism that allows bacteria to survive during antibiotic treatment (41). However, reducing the bacterial ATP level with an inhibitor, such as BDQ, below a threshold concentration causes rapid cell death (9, 42). In addition, another study reported that a rapid increase in cellular ATP is related to the formation of reactive oxygen species (ROS), which are a key component in the induction of bacterial cell death (36, 43). Based on metabolic analysis of E. coli, the number of ROS-generating reactions is comparable to the number of reactions that generate ATP/ADP, NAD/H, and NADP/H, and metabolic reconstruction revealed that energy generation and energy consumption are the major sources accounting for ROS production (44). Tatano and colleagues previously demonstrated that supplementation with exogenous ATP exerted antimycobacterial activity, which further indicated the cytotoxic effect of ATP on the upper threshold concentration (45). In summary, cellular ATP homeostasis is critical for bacterial viability, and ATP fluctuations are a key paradigm for studying the mechanism behind the cellular stress response and energy metabolism.

Besides environmental stress, we also observed fluctuations in cellular ATP levels of various magnitudes following treatment with antibiotics (18). Pioneering research revealed the link between bacterial metabolism and antibiotics, showing that bacteriostatic drugs tend to decrease the respiration rate while antibiotics with bactericidal activity tend to increase the bulk oxygen consumption rate, indicating that antibiotics have various impacts on cellular energy generation (25, 37). Additionally, anti-TB drugs cause ATP switching, with different kinetic profiles, in M. smegmatis, and loss of bioenergetic homeostasis is correlated with an increase in drug susceptibility for M. tuberculosis (46), promoting the killing effect of antibiotics. Consistent with our study, these studies illustrated that the efficacy of antibiotics is strongly correlated with perturbation of the cellular ATP level, emphasizing that the bacterial ATP level is an indicator of antibiotic efficacy.

Our data demonstrated that the antibiotic efficacy of drug combinations in which the components have opposite effects on energy metabolism is attenuated. An electron transport chain inhibitor attenuated the efficacy of INH and moxifloxacin (MXF). INH and MXF can both induce mycobacterial cell death due to a transient increase in intracellular ATP (47). Our research further confirmed that drug combinations consisting of components altering mycobacterial ATP levels can similarly promote the perturbation of ATP levels, resulting in the enhanced efficacy of chemotherapeutics. Considering the close relationship between mycobacterial ATP fluctuations and antibiotic-induced cell death, cellular ATP levels might be a good indicator with which to develop potent drug combinations for the treatment of TB. Drug combination is an effective strategy for the treatment of clinical infections, especially for preventing antibiotic resistance (48). Clinical trials and regression analysis are needed for drug combination design, where broth-based checkerboard assays and time-kill experiments are the gold standards. However, case-by-case examinations of antibiotic interactions and patient isolates are needed to determine the additive, synergistic, or antagonistic reactions between different drugs. Several strategies have been developed to test antibiotic synergy more efficiently, such as the CombiANT assay. However, special three-dimensional (3D) printed agar plates are required for this approach (49). Utilizing the ATPser, we found that BDQ and INH had opposite effects on cellular ATP alternation, which were accompanied by antagonistic reactions for the killing of mycobacteria. Recently, a sampling and scoring method (DiaMOND) clarified the weak antagonistic relationship between BDQ and INH (50). In our study, INH and EMB both increased mycobacterial ATP levels, and the combination of these two drugs exhibited additive/synergistic antibiotic effects. In fact, EMB induces repression of the envelope stress-related gene *inhA* in mycobacteria and thereby enhances the antibiotic effect of INH; therefore, this chemotherapeutic combination is effective for the treatment of TB (38). These results highlighted the important role of cellular ATP levels in drug combination design, and potentially efficient drug combinations can be conceived based on their similar effects on bacterial ATP.

However, challenges also remain in drug combination design based on cellular ATP fluctuations. For example, it has been reported that antibiotics can be classified according to whether they are strongly or weakly dependent on metabolism, based on their effect on cellular ATP levels (51). However, not all antibiotics rely on cellular metabolism fluctuations as part of their killing mechanism, which may limit the application of ATPser. In addition, different strains exhibit different levels of sensitivity toward cellular ATP perturbation. Treatment of Mycobacterium bovis BCG vaccine strain with the oxidative phosphorylation inhibitor CCCP significantly reduced cellular ATP levels but had no influence on viability (22). Furthermore, Turapov and colleagues verified that CCCP treatment promoted the generation of persister cells for BCG under nonpermissive growth conditions and increased bacterial viability (52). However, Chen and coworkers reported that CCCP exhibits direct antibacterial activity against Mycobacterium abscessus (53). They also found that the CCCP MIC differed among different Mycobacterium strains, explaining why sensitivity toward CCCP varies among strains. Therefore, the correlation between cellular ATP fluctuations and antibiotic efficacy is determined not only by the drug class but also by the bacterial strain.

In summary, we developed a single-fluorescence FRET-based ATP biosensor that was applied to mycobacteria for the convenient measurement of cellular ATP fluctuations caused by environmental stress and antibiotics. Using this tool, we clarified that ATP perturbation is a crucial indicator for the efficacy of antibiotic regimes. ATPser may provide useful guidance for drug combination design in clinical practice.

## MATERIALS AND METHODS

### Bacterial strains and culture conditions.

M. smegmatis mc^2^ 155, which is an efficient plasmid transformation mutant of M. smegmatis ATCC 607 first isolated by Snapper et al. (54, 55) with a point mutation in the gene encoding the SMC (structural maintenance of chromosomes) protein (56), was grown at 37°C in Middlebrook 7H9 broth supplemented with 0.5% glycerol (M. tuberculosis), 0.05% Tween 80, and 10% oleic acid-albumin-dextrose-catalase (OADC) or in Luria-Bertani (LB) solid medium. E. coli strain DH5α was utilized for plasmid cloning and was cultivated at 37°C in LB broth or agar plate. M. tuberculosis mc^2^ 6206 was cultured in Middlebrook 7H9 broth or on 7H10 agar medium supplemented with OADC, 0.2 to 0.5% glycerol, 0.05% tyloxapol, and required supplements for the double auxotroph mc^2^ 6206, including 0.2% Casamino Acids, pantothenic acid (24 μg/mL), and leucine (50 μg/mL).

### Construction of mycobacterial ATP reporter strain.

To indicate the intracellular ATP level fluctuation, ATPser (ATP sensor) DNA sequence had been optimized for expression in mycobacteria and was inserted into a mycobacterial integrating vector [pRH2502 (57), a pMV306 derivative with kanamycin resistance] to obtain pATPser (see Fig. S1 in the supplemental material). Briefly, pRH2502 was restricted by NdeI*/*PacI (Thermofisher; FastDigest) for linearization. The inserted DNA fragment encoding ATPser was then amplified by PCR with primers iATPSnFr-NdeI-5F and iATPSnFr-PacI-3R. Both primers contain 15- to 20-bp overlapping arms homologous with the 3′ end of the restricted plasmid. The purified plasmid and target fragment were mixed at a molar ratio of 1:3, 2× ClonExpress mix (ClonExpress Ultra one-step cloning kit; Vazyme) was added to the reaction mixture, and the sample was incubated in a thermocycler at 50°C for 15 min. The target fragment was inserted into the restricted plasmid through homologous recombination with three enzymes in the mix: a 5′ exonuclease generates long overhangs, a polymerase fills in the gaps of the annealed single-stranded regions, and a DNA ligase seals the nicks of the annealed and filled-in gaps. Finally, the well-reacted product was directly transformed into E. coli DH5-α competent cells for enrichment. As a result, all the plasmid constructs were confirmed via Sanger sequencing.

10.1128/msystems.00209-22.1 FIG S1 Plasmid map of pATPser encoding ATPser in M. smegmatis mc^2^ 155. The plasmid map exhibits the construction and elements of pATPser, which was based on the plasmid backbone of pRH2502. *aph*, kanamycin phosphotransferase; *int*, integrase from phage L5; *attB*, site for integration at *attP* of the mycobacterial genome; *uv15tet0*, Pmyc1tetO promoter; iATPSnFr, gene encoding ATPser; pBR322 origin, origin of replication in E. coli. The plasmid map was plotted by SnapGene (version 5.2). Download FIG S1, TIF file, 1.0 MB. Copyright © 2022 Liang et al. 2022 Liang et al. https://creativecommons.org/licenses/by/4.0/ This content is distributed under the terms of the Creative Commons Attribution 4.0 International license.

### Construction of mycobacterial ClpB-EGFP reporter strain.

Previous studies showed that the formation of polar protein aggresomes is related to the cellular stress response and the disaggregation of aggresomes requires the assistance of ClpB, which gathers at two poles in the stress response cell (20, 34). We therefore labeled mycobacterial ClpB with EGFP to indicate the cellular stress response. The ClpB-EGFP reporter strain was generated as previously described (58, 59). Briefly, two homologous arms, approximately 500 bp, flanking the ClpB stop codon were obtained through PCR and cloned at the borders of the EGFP-hygromycin excisable cassette (primer pairs ClpB-UP-5F and ClpB-UP-3R for amplification of the ClpB upstream arm, primer pairs ClpB-DO-5F and ClpB-DO-3R for amplification of the ClpB downstream arm, and primer pairs ClpB-GFP-5F and ClpB-GFP-3R for amplification of the EGFP-hygromycin cassette). The donor DNA fragment comprised an upstream homologous arm, *egfp*, a gene coding for the hygromycin excisable cassette, and a downstream homologous arm, which was purified with FastPure DNA mini columns (Vazyme) and electroporated into the competent cells of M. smegmatis harboring pJV53 plasmid (60). The hygromycin-resistant colonies were grown for several generations in the absence of hygromycin and kanamycin to allow excision of the hygromycin cassette and loss of pJV53. Finally, the unmarked ClpB-EGFP strain was confirmed by PCR and sequencing (Fig. S2).

10.1128/msystems.00209-22.2 FIG S2 Construction of the ClpB-EGFP reporter strain. (A) A schematic representation of the strategy used for generating the WT_ClpB_EGFP reporter strain is shown. Confirmation of the WT_ClpB_EGFP reporter strain was performed by PCR amplifications with specific primers. (B) PCR amplification yielded a product band of 1.2 kb for the WT_ClpB_EGFP reporter strain but not for wild-type M. smegmatis. Download FIG S2, TIF file, 0.1 MB. Copyright © 2022 Liang et al. 2022 Liang et al. https://creativecommons.org/licenses/by/4.0/ This content is distributed under the terms of the Creative Commons Attribution 4.0 International license.

### Bulk measurement of intracellular ATP level.

A CellTiter-Glo 2.0 assay kit (Promega) was used for bulk measurement of the mycobacterial cellular ATP level. Aliquots (100 μL) of WT_ATPser culture were collected, mixed with an equal volume of CellTiter-Glo reagent in a 96-well, half-area-black, opaque plate, and allowed to react at room temperature for 10 min in the dark with shaking. The luminescence signals were measured with a microplate reader (BioTek). The background signal was identified by performing the same procedures on aliquots of medium without bacteria, and the background signal for each sample was removed from the total signal. The ATP standard curve was assayed in parallel for the calculation of the bulk ATP concentration. Meanwhile, the cell numbers of each sample for testing were verified by a CFU assay, and the ATP concentration of each individual cell was verified as the bulk ATP concentration/CFU. Three replicates were used for each experiment.

### Fluorescent imaging and single-cell signal processing.

The fluorescent signals for WT_ATPser were measured as previously described (21). Briefly, after treatment with different antibiotics or stress, the samples of WT_ATPser were subjected to fluorescence microscopy for detecting signals of 488 nm_ex_ and 405 nm_ex_. Fluorescein isothiocyanate (FITC) at 488 nm excitation (488 nm_ex_) and 4′,6-diamidino-2-phenylindole (DAPI) at 405 nm excitation (405 nm_ex_) were recorded by Olympus BX63 fluorescence microscopy on a phase-contrast microscope equipped with a 100× oil immersion objective and a xenon lamp. Exposure time and gaining, respectively, were 425 ms and 1× for phase contrast, 1.2 s and 4× for 488 nm_ex_, and 625 ms and 4× for 405 nm_ex_. All experiments were repeated three times with similar results for approximately 100 isolates. The collected images were transformed into 16-bit TIF format and processed by Fiji software. The process for calculating the 488 nm_ex_/405 nm_ex_ ratio was as follows: the target signal for each individual cell within the same image was selected by adjusting the threshold and creating a region of interest (ROI). The noise outside the ROI was removed for both the 488 nm_ex_ and 405 nm_ex_ images. For the same view, the 488 nm_ex_/405 nm_ex_ ratio of each cell was obtained by image division and is represented by a 16-color lookup table, and the color scale bar for the ratio values indicates high and low intracellular ATP levels. The final images were exported in the red-green-blue (RGB) color format. Also, the fluorescent density of each particle within the same view was selected as the ROI by adjusting the threshold and measured as the gray value by Fiji.

### Determination of ClpB focus ratio.

The fluorescent signals for WT_ClpB_EGFP were collected as described in previous research (61). In brief, FITC 488-nm excitation (488 nm_ex_) was recorded by Olympus BX63 fluorescence microscopy on a phase-contrast microscope equipped with a 100× oil immersion objective and a xenon lamp. Exposure time and gaining, respectively, were 425 ms and 1× for phase contrast and 1.7 s and 4× for 488 nm_ex_. All experiments were repeated three times with similar results for approximately 100 isolates. The collected images were transformed into 16-bit TIF format and processed by Fiji software. The fluorescent signal from 488 nm_ex_ was false colored in green. The isolates displaying ClpB-EGFP focus were considered dormant cells, and the ratio of persisters is the number of isolates displaying ClpB-EGFP foci/total cell number. All experiments were repeated three times with similar results for approximately 100 isolates.

### Drug treatment.

M. smegmatis mc^2^ 155 WT_ATPser was grown to mid-exponential phase and treated separately with several antibiotics for 4 h as follows: 0.025 μg/mL (0.5× MIC), 0.05 μg/mL (1× MIC), 0.5 μg/mL (10× MIC), 5 μg/mL (100× MIC), 10 μg/mL (200× MIC), and 20 μg/mL (400× MIC) bedaquiline (BDQ); 4 μg/mL (0.5× MIC), 8 μg/mL (1× MIC), 80 μg/mL (10× MIC), and 800 μg/mL (100× MIC) rifampin (RIF); 4 μg/mL (0.5× MIC), 8 μg/mL (1× MIC), 80 μg/mL (10× MIC), and 800 μg/mL (100× MIC) isoniazid (INH); and 1, 10, and 100 μM CCCP. Also, M. tuberculosis mc^2^ 6206 carrying pATPser was treated with antibiotics for 12 h as follows: 0.0823 μg/mL (1× MIC), 0.823 μg/mL (10× MIC), and 8.23 μg/mL (100× MIC) rifampin (RIF); 0.28 μg/mL (1× MIC), 2.8 μg/mL (10× MIC), and 28 μg/mL (100× MIC) bedaquiline (BDQ); 0 μg/mL (control), 0.5 μg/mL (1× MIC), 5 μg/mL (10× MIC), and 50 μg/mL (100× MIC) isoniazid (INH). After treatment, the cells were collected by centrifugation and washed twice with saline. The sample without antibiotic treatment was regarded as the untreated control. The fluorescent signals were verified by fluorescence microscopy, and the viability was determined by the CFU assay. All experiments were repeated three times.

### Starvation treatment.

M. smegmatis mc^2^ 155 WT_ATPser was grown to mid-exponential phase, and cells were collected by centrifugation at 4,000 rpm for 3 min and washed twice with saline, followed by resuspension with the same volume of PBST. The culture was continuously cultivated with shaking, and samples were collected 0 h, 24 h, 48 h, and 72 h after starvation treatment. The fluorescent signals were verified by fluorescence microscopy, and the viability was determined by the CFU assay. All experiments were repeated three times.

### ATP synthesis inhibitor CCCP treatment.

M. smegmatis mc^2^ 155 WT_ATPser was grown to mid-exponential phase and treated with 1, 10, and 100 μM CCCP (carbonyl cyanide *m*-chlorophenyl hydrazone) for 4 h. After treatment, the cells were collected by centrifugation at 4,000 rpm for 3 min and washed twice with saline. The sample without antibiotic treatment was regarded as the untreated control. The fluorescent signals were verified by fluorescence microscopy, and the viability was determined by the CFU assay. All experiments were repeated three times.

### Measurement of growth curve.

The growth curves of M. smegmatis mc^2^ 155 expressing ATPser (WT_ATPser) and M. smegmatis mc^2^ 155 carrying pMV306 empty plasmid (M. smegmatis WT) as the control were measured as followed: fresh single colonies of the target strains were inoculated in 7H9 medium containing 25 μg/mL kanamycin (Kan) separately and cultivated overnight at 37°C. The cultures were then adjusted to an optical density at 600 nm (OD_600_) of 0.5 with saline and diluted at a ratio of 1:10. For each strain, 20 μL of diluted culture was added to 180 μL of 7H9 medium containing 25 μg/mL Kan in a 96-well plate. A microplate reader (BioTek) was used to measure the optical density of each well every 3 h. Three replicates were used for each strain. The growth curve was plotted by RStudio (version 3.6.1). Meanwhile, the growth rates for exponential phase (9 to 45 h) of the two strains were also calculated. The growth rate between two time points was calculated with the algorithm *k*(growth rate) = (ln*W_2_ −* ln*W_1_*)/(*t_1_ − t_2_*), where *W* is the OD_600_ value for each time point. The average represents the growth rate during exponential phase.

### Drug combination treatment.

The M. smegmatis mc^2^ 155 WT_ATPser reporter strain was treated with the following drug combinations: BDQ+CCCP, EMB+INH, and BDQ+INH. All the drugs were 10× MICs in concentration (0.5 μg/mL BDQ, 80 μg/mL RIF, 80 μg/mL INH, and 80 μg/mL EMB), and the concentration for CCCP was 10 μM. After treatment, the cells were collected by centrifugation at 4,000 rpm for 3 min and washed twice with saline. The sample without antibiotic treatment was regarded as the untreated control. The fluorescent signals were verified by fluorescence microscopy as described above, and the viability was determined by the CFU assay. All experiments were repeated three times.

### CFU assay.

For viable CFU counting after drug treatment, 100 μL each of 1:10^4^ and 1:10^6^ diluted culture was plated on an LB agar plate containing kanamycin. After incubation at 37°C for 72 h, viable CFU were counted.

### Statistical analysis.

Statistical analysis was performed using Prism (version 8.0c; GraphPad Software). Data were analyzed using the paired Student *t* test, and in the comparisons of data from three or more conditions, analysis of variance (ANOVA) was used. A *P* value of 0.05 or less was considered statistically significant. Correlation analysis was performed using RStudio (version 3.6.1).

10.1128/msystems.00209-22.8 TABLE S1 Bacterial strains or plasmids used in this study. All the bacterial strains, plasmids, and primers used in this research are given. Download Table S1, PDF file, 0.2 MB. Copyright © 2022 Liang et al. 2022 Liang et al. https://creativecommons.org/licenses/by/4.0/ This content is distributed under the terms of the Creative Commons Attribution 4.0 International license.

10.1128/msystems.00209-22.9 TABLE S2 ATPser construct sequences. The sequences of nucleic acids encoding ATPser are given. Download Table S2, DOCX file, 0.02 MB. Copyright © 2022 Liang et al. 2022 Liang et al. https://creativecommons.org/licenses/by/4.0/ This content is distributed under the terms of the Creative Commons Attribution 4.0 International license.
