# Supplementary figures and images for: Single-Fluorescence ATP Sensor Based on Fluorescence Resonance Energy Transfer Reveals Role of Antibiotic-Induced ATP Perturbation in Mycobacterial Killing
Source: mSystems. 2022 May 26;7(3):e00209-22. doi: 10.1128/msystems.00209-22 (PMC9238375; doi:10.1128/msystems.00209-22)

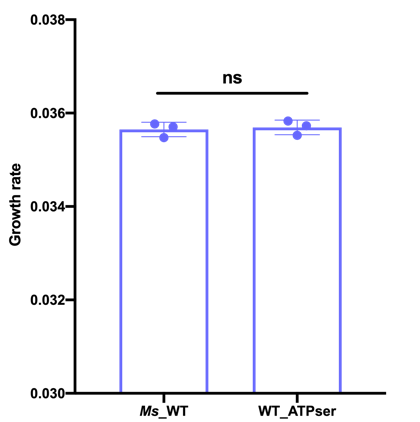

Supplement: FIG S3 [file msystems.00209-22-s0003.tif]

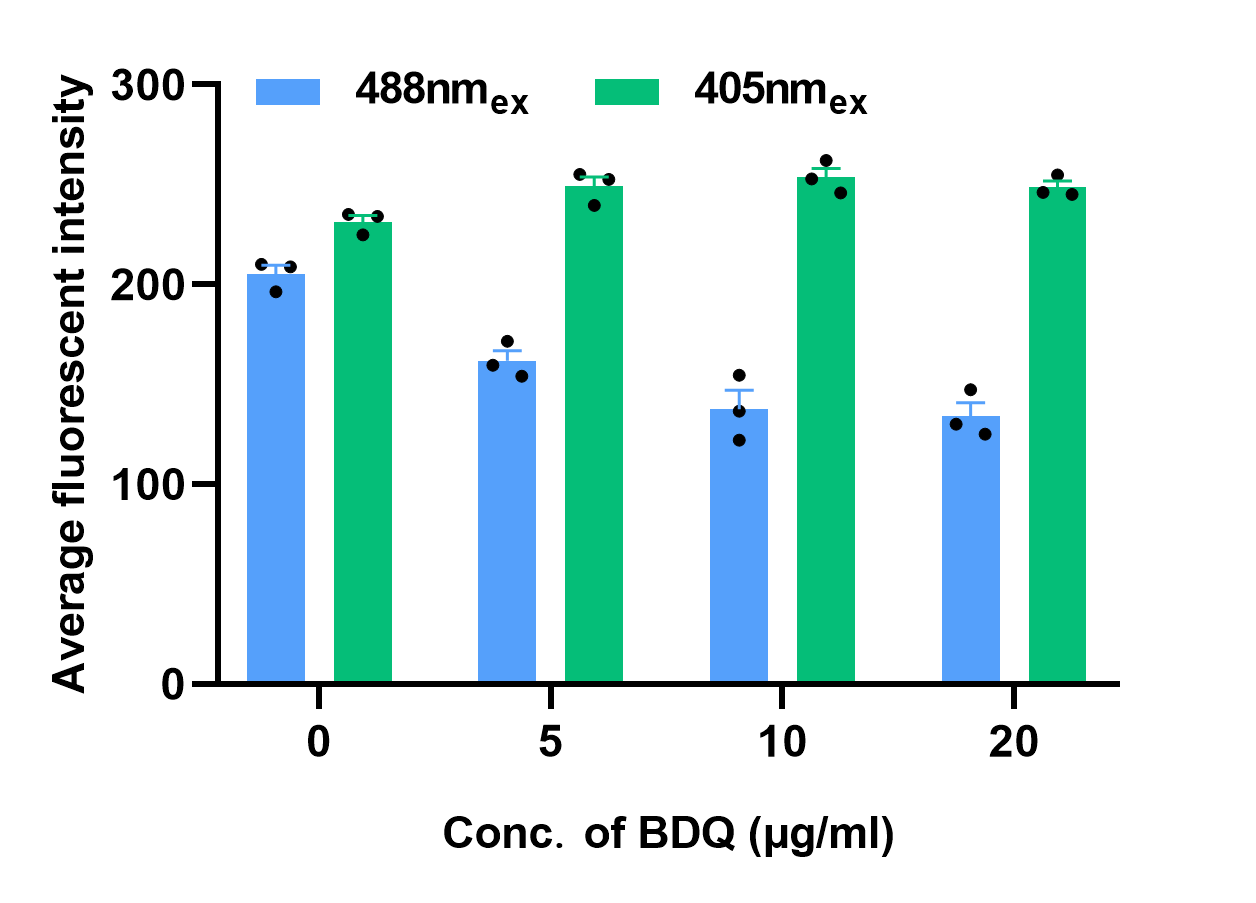

Supplement: FIG S4 [file msystems.00209-22-s0004.tif]

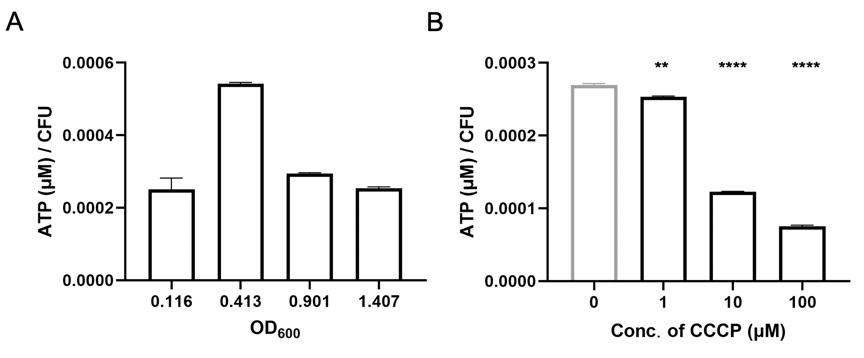

Supplement: FIG S5 [file msystems.00209-22-s0005.tif]

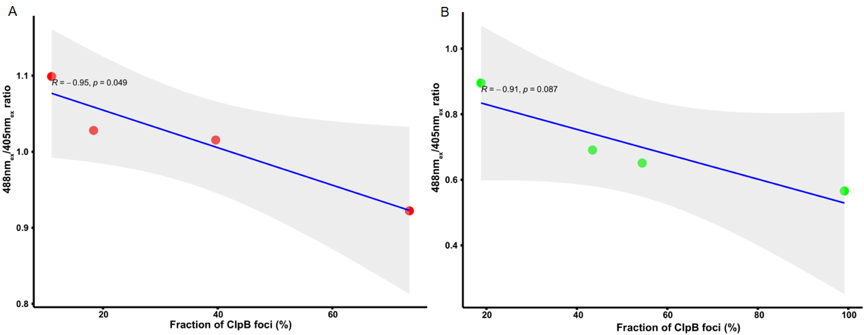

Supplement: FIG S6 [file msystems.00209-22-s0006.tif]

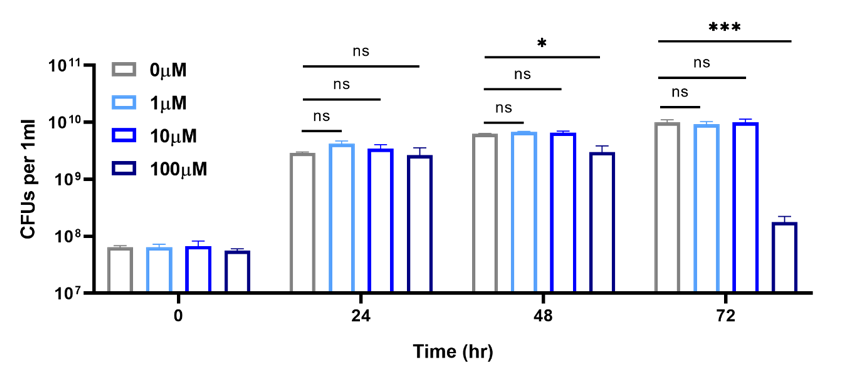

Supplement: FIG S7 [file msystems.00209-22-s0007.tif]

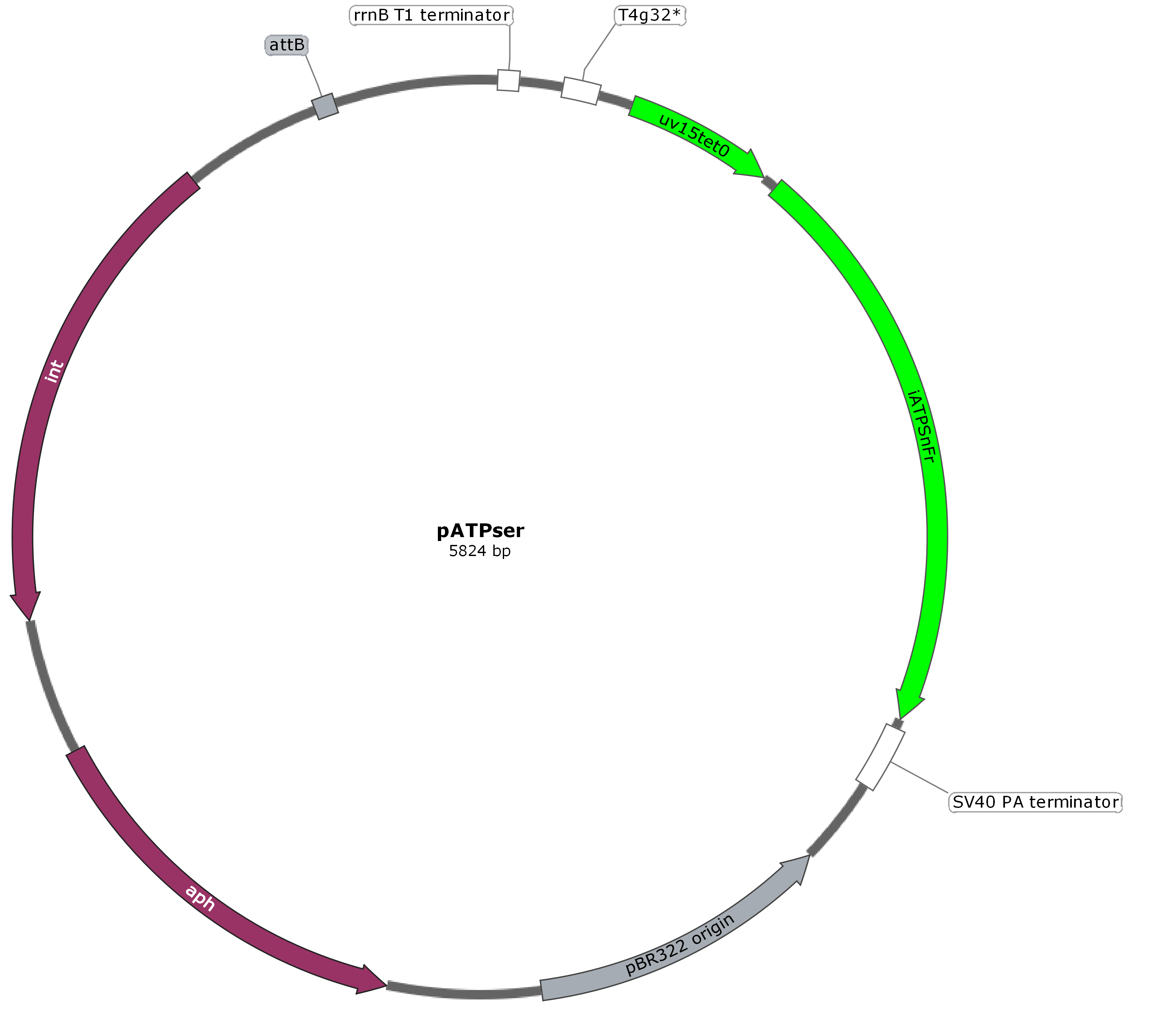

Supplement: FIG S1 [file msystems.00209-22-s0001.tif]

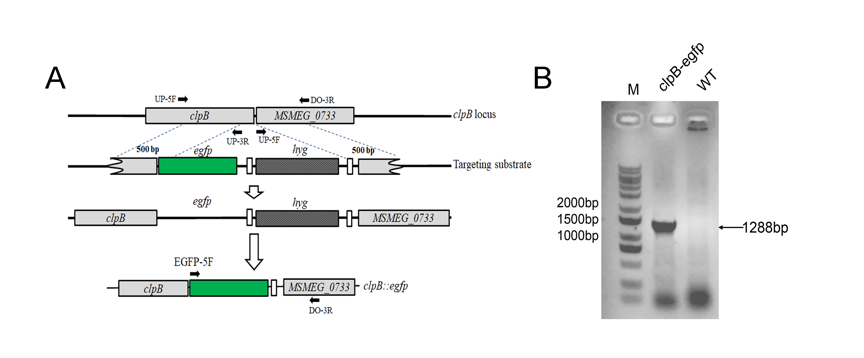

Supplement: FIG S2 [file msystems.00209-22-s0002.tif]
