# Supplementary material for: Single-Fluorescence ATP Sensor Based on Fluorescence Resonance Energy Transfer Reveals Role of Antibiotic-Induced ATP Perturbation in Mycobacterial Killing
Source: mSystems. 2022 May 26;7(3):e00209-22. doi: 10.1128/msystems.00209-22 (PMC9238375; doi:10.1128/msystems.00209-22)
Supplement: TABLE S1 [file msystems.00209-22-s0008.pdf]

Table S1. Bacterial strains or plasmids used in this study

Bacterial strains or plasmids	Description	Reference/source
**strains**		
WT_*Ms*	Wild type *Mycobacterium smegmatis* mc2 155	Our lab
WT_ ATPser	*M. smegmatis* containing ATPser	this study
WT_*ClpB-EGFP*	*M. smegmatis* with EGFP label at ClpB protein	this study
E.coli DH5α	F-, φ80d lacZ ΔM15, Δ(lacZYA argF) U169, deoR , recA1 , endA1 , hsdR17 (rK-, mK+), phoA, supE44 , λ-, thi-1, gyrA96 , relA1	Our lab
M. tuberculosis mc^{2} 6206	*M. tuberculosis* H37Rv *ΔpanCD ΔleuCD auxotroph*	Our lab
**Plasmids**		
pJV53	Che9c genes 60–61 under control of the acetamidase promoter in pLAM12 . KanR	Addgene #26904
pRH2502	Mycobacterial integrating vector pTC-0X-1L derivative, KanR	Addgene #84379
pATPser	Mycobacterial integrating vector pTC-0X-1L derivative containing ATPser, KanR	
**Primers**		
iATPSnFr-NdeI-5F	TGCGAAGGAGATATACATATGATGAAGACCATCCACGTCTCGG	this study
iATPSnFr-PacI-3R	CAAGTTCCGAGTTTGTTAATTAATCAGTTCAGGTCTTCCTCCGAG	this study
ClpB-UP-5F	CTACATCGGTTACGACCAGG	this study
ClpB-UP-3R	TCCTCGCCCTTGCTCACCATGCCCAAAACGAGGGACTCGC	this study
ClpB-GFP-5F	GCGAGTCCCTCGTTTTGGGCATGGTGAGCAAGGGCGAGGA	this study
ClpB-GFP-3R	ATGCCGTTTGGCGCTGGTAGAAGATCTTTACTTGTACAGCTCGTCC	this study
ClpB-DO-5F	GGACGAGCTGTACAAGTAAAGATCTTCTACCAGCGCCAAACGGCAT	this study
ClpB-DO-3R	GTACACGCGCGTGTTCGCCG	this study
