# Supplementary material for: Single-Fluorescence ATP Sensor Based on Fluorescence Resonance Energy Transfer Reveals Role of Antibiotic-Induced ATP Perturbation in Mycobacterial Killing
Source: mSystems. 2022 May 26;7(3):e00209-22. doi: 10.1128/msystems.00209-22 (PMC9238375; doi:10.1128/msystems.00209-22)
Supplement: TABLE S2 [file msystems.00209-22-s0009.docx]

Table S2: ATPser construct sequences

| ATGAAGACCATCCACGTCTCGGTGGTGACCCCGGACGGCCCGGTCTACGAGGACGACGTGGAGATGGTCAGCGTGAAGGCCAAGAGCGGCGAGCTGGGCATCCTGCCGGGCCACATCCCGCTGAAGGCGCCGCTGGAGATCTCGGCGGCCCGGCTGAAGAAGGGCGGCAAGACCCAGTACATCGCGGTCTCGGGCGGCAACCTGGAGGTCCGCCCGGACAAGGTGACCATCTACGCCCAGGCGGCCGAGCGGGCCGAGGACATCGACGTGCTGCGGGCCAAGGCGGCCAAGGAGCGGGCGGAGCGGCGACTGCAGAGCCAGGTCCTGTCGCACAACGTGTACATCACCGCCGACAAGCAGAAGAACGGCATCAAGGCGAACTTCAAGATCCGGCACAACGTCGAGGACGGCTCGATGCAGCTGGCGGACCACTACCAGCAGAACACCCCGATCGGCGACGGCCCGGTCCTGCTGCCGGACAACCACTACCTGTCGACCCAGAGCGTGCTGTCGAAGGACCCGAACGAGAAGCGCGACCACATGGTCCTGCTGGAGTTCGTGACCGCGGCCGGCATCACCCTGGGCATGGACGAGCTGTACAAGGGCGGCACCGGCGGCAGCATGAGCAAGGGCGAGGAGCTGTTCACCGGCGTGGTGCCGATCCTGGTGGAGCTGGACGGCGACGTGAACGGCCACAAGTTCTCGGTGCGGGGCGAGGGCGAGGGCGACGCCACCAACGGCAAGCTGACCCTGAAGTTCATCTGCACCACCGGCAAGCTGCCGGTGCCGTGGCCGACCCTGGTGACCACCCTGACCTACGGCGTCCAGTGCTTCTCGCGCTACCCGGACCACATGAAGCAGCACGACTTCTTCAAGTCGGCCATGCCGGAGGGCTACGTGCAGGAGCGCACCATCTCGTTCAAGGACGACGGCACCTACAAGACCCGGGCGGAGGTCAAGTTCGAGGGCGACACCCTGGTGAACCGCATCGAGCTGAAGGGCATCGACTTCAAGGAAGACGGCAACATCCTGGGCCACAAGCTGGAGTACAACTTCGGCCTGCACGACATCGACTTCAAGCGGGCGGAGCTGGCCCTGAAGCGGGCCATGAACCGGCTGTCGGTGGCCGAGATGAAGCTGCAGGTGGACGAGCAGAAGCTGATCTCGGAGGAAGACCTGAACTGA |
| --- |
